# Nuclear Localization Sequence of FGF1 Is Not Required for Its Intracellular Anti-Apoptotic Activity in Differentiated Cells

**DOI:** 10.3390/cells11030522

**Published:** 2022-02-02

**Authors:** Agata Lampart, Katarzyna Dominika Sluzalska, Aleksandra Czyrek, Aleksandra Szerszen, Jacek Otlewski, Antoni Wiedlocha, Malgorzata Zakrzewska

**Affiliations:** 1 Department of Protein Engineering, Faculty of Biotechnology, University of Wroclaw, ul. F. Joliot-Curie 14a, 50-383 Wroclaw, Poland; agata.lampart@uwr.edu.pl (A.L.); katarzyna.sluzalska@uwr.edu.pl (K.D.S.); aszerszen00@gmail.com (A.S.); jacek.otlewski@uwr.edu.pl (J.O.); 2 Department of Protein Biotechnology, Faculty of Biotechnology, University of Wroclaw, ul. F. Joliot-Curie 14a, 50-383 Wroclaw, Poland; aleksandra.czyrek@uwr.edu.pl; 3 Department of Molecular Cell Biology, Institute for Cancer Research, The Norwegian Radium Hospital, Oslo University Hospital, Montebello, 0379 Oslo, Norway; antoni.wiedlocha@ous-research.no; 4 Centre for Cancer Cell Reprogramming, Institute of Clinical Medicine, Faculty of Medicine, University of Oslo, Montebello, 0379 Oslo, Norway

**Keywords:** FGF1, translocation, intracellular FGF1, nuclear FGF, nuclear localization sequence, anti-apoptotic activity

## Abstract

Fibroblast growth factor 1 (FGF1) is considered primarily as a ligand for FGF surface receptors (FGFRs) through which it activates a number of cellular responses. In addition to its canonical mode of action, FGF1 can act intracellularly, before secretion or after internalization and translocation from the cell exterior. The role of FGF1 inside the cell is to provide additional protection against apoptosis and promote cell survival. The FGF1 protein contains a specific N-terminal nuclear localization sequence (NLS) that is essential for its efficient transport to the nucleus. Here, we investigated the role of this sequence in the anti-apoptotic response of FGF1. To this end, we produced recombinant FGF1 variants with mutated or deleted NLS and added them to apoptosis-induced cells in which FGFR1 was inactive, either as a result of chemical inhibition or kinase-dead mutation. After internalization, all FGF1 variants were able to protect the differentiated cells from serum starvation-induced apoptosis. To verify the results obtained for NLS mutants, we knocked down LRRC59, a protein that mediates the nuclear transport of FGF1. Upon LRRC59 silencing, we still observed a decrease in caspase 3/7 activity in cells treated exogenously with wild-type FGF1. In the next step, FGF1 variants with mutated or deleted NLS were expressed in U2OS cells, in which apoptosis was then induced by various factors (e.g., starvation, etoposide, staurosporine, anisomycin and actinomycin D). Experiments were performed in the presence of specific FGFR inhibitors to eliminate FGFR-induced signaling, potentially activated by FGF1 proteins released from damaged cells. Again, we found that the presence of NLS in FGF1 is not required for its anti-apoptotic activity. All NLS variants tested were able to act as wild type FGF1, increasing the cell viability and mitochondrial membrane potential and reducing the caspase 3/7 activity and PARP cleavage in cells undergoing apoptosis, both transiently and stably transfected. Our results indicate that the nuclear localization of FGF1 is not required for its intracellular anti-apoptotic activity in differentiated cells and suggest that the mechanism of the stress response differs according to the level of cell differentiation.

## 1. Introduction

Fibroblast growth factor 1 (FGF1) acts through specific interactions with surface FGF receptors (FGFRs) to activate intracellular signaling axes, such as PLCγ/PKC, PI3K/Akt, and Ras/MAPK, regulating cell growth, proliferation, survival, and playing a significant role in development [1,2,3]. In addition to this canonical modus operandi, FGF1 has the unique ability to translocate across cell membranes, reaching the cytosol and then nucleus [4,5,6,7,8]. In the early 1990s, Imamura and coworkers discovered that FGF1 has a specific N-terminal nuclear localization sequence (NLS), which is essential for efficient transport of FGF1 to the nucleus and its mitogenic activity [7,8]. Interestingly, the selected monopartite sequence NYKKPKL (residues 21 to 27) was similar to the NLSs of other nuclear proteins such as c-myb, N-myc, p53 and c-erb-A [9,10]. However, later studies have shown that deletion of this signal sequence results in loss of the protein stability, protease resistance and reduced affinity for FGFRs [11], which could explain it impaired biological activity. When point mutations were introduced in the NLS of FGF1, they do not affect FGF1 ability to stimulate mitogenic response [12,13].

Over the years, reports of evidence for nuclear translocation of FGF1 began to emerge, laying the groundwork for elucidating its mechanism. Stress conditions including serum starvation, oxidative stress, and hyperosmolarity have been shown to promote FGF1 translocation in the G1 phase of the cell cycle [14,15,16]. Only two FGF receptors (FGFR1 and FGFR4) are able to mediate this process [15].Moreover, the presence of a specific sequence within the C-terminus of the receptor is required for the FGF1 translocation, whereas the active kinase domain of the receptor appears to be irrelevant in thisprocess [15,17,18]. Despite ongoing efforts, the exact fate of the FGF1-FGFR complex when crossing the endosomal membrane, as well as the timing of its dissociation, is not yet fully understood.

Nevertheless, it is known that vesicular membrane potential is required for FGF1 translocation from early endosomes [19], and numerous cytosolic molecules such as PI3K [20], HSP90 [21] and p38 kinase [16] are involved in this process. After leaving the endosomes, FGF1 can interact with conventional transport proteins including karyopherin alpha1, karyopherin beta1, exportin-1, Ran, and LRRC59 [22,23,24,25]. Of note, interaction with LRRC59 [22] is critical for the nuclear import of exogenous FGF1 [26].

About six hours after FGFR binding, the amount of FGF1 translocated into the nucleus is maximal. In the nucleus, PKCδ phosphorylates FGF1 at serine 130, providing a signal for FGF1 export back to the cytosol and subsequent degradation [27]. In addition, FGF1 also contains a leucine-rich nuclear export sequence (NES), which is responsible for its nuclear export [25].

In 2005, Wesche et al. described a second NLS of FGF1, a bipartite one characterized by two lysines clusters separated by 10 AA. Moreover, both of them are required for FGF1 transport into the nucleus. Several mutations involving the substitution of lysines to alanines were designed. Variants with mutated N-terminal NLS and first cluster of bipartite NLS after addition to NIH 3T3 cells were found in the cytosolic but not in the nuclear fraction [13].

Although the trafficking of internalized FGF1 has been described in some detail, much remains to be uncovered in understanding its intracellular functions. We have recently shown that translocation of exogenous FGF1 protects cells from apoptosis through a pathway independent of FGFR signaling [28]. FGF1, which after internalization crosses the endosomal membrane, exerts an anti-apoptotic effect regardless of receptor activation.

Many studies have also focused on elucidating the neurotrophic and pro-nutritional properties of FGF1. Ectopic expression of FGF1 has been shown to promote neuronal differentiation [29] and inhibit p53-dependent apoptosis in undifferentiated PC12 cells [30,31]. The same group suggested that these features are regulated by C-terminal domain of FGF1 and its phosphorylation [32]. Importantly, deletion of the N-terminal NLS motif of FGF1 resulted in diminished neuronal differentiation and protection from p53-dependent apoptosis [33]. As the stability of the ΔNLS variant is arguable, further analyses are required to fully elucidate the role of NLS in the FGF1 function.

Here, we have investigated the role of the NLS motifs in the anti-apoptotic response generated by FGF1. We found that both endogenously and exogenously administrated NLS variants did not show reduced anti-apoptotic activity compared with the wild type in differentiated cells. Even upon LRRC59 silencing, we have still observed a protective effect of FGF1. Our results thus indicate that the nuclear localization of FGF1 is not essential for its intracellular anti-apoptotic activity in differentiated cells.

## 2. Materials and Methods

### 2.1. Antibodies and Reagents

Unless otherwise indicated, all reagents were purchased form Merck (Darmstadt, Germany). The following primary antibodies were used: goat anti-FGF1 (sc-1884), mouse anti-FGF1 (sc-55520), and mouse anti-FGFR4 (sc-136988) from Santa Cruz Biotechnology (Dallas, TX, USA); mouse anti-γ-tubulin (T6557) and rabbit anti-LRRC59 (HPAA030829) from Sigma-Aldrich (Darmstadt, Merck, Germany); rabbit anti-PARP (9542), rabbit anti-pERK1/2 (9101), rabbit anti-ERK1/2 (9102), rabbit anti-FGFR1 (9740), rabbit anti-FGF1 (3139), and rabbit anti-FGFR2 (11835) from Cell Signaling Technology (Danveres, MA, USA); goat anti-FGF1 (AF232) from R&D Systems (Minneapolis, MN, USA); mouse anti-lamin A (ab8980)and rabbit anti-FGFR3 (ab10559) from Abcam (Cambridge, UK); rabbit anti-pFGFR1 (06-1433) from Merck (Darmstadt, Germany). Rabbit polyclonal anti-FGF1 antibodies (OMA1) were generated by Davids Biotechnologie GmbH (Regensburg, Germany) by immunization of rabbits with purified FGF1. Secondary antibodies coupled to HRP: donkey anti-mouse (115-035-003), donkey anti-rabbit (111-035-144) and donkey anti-goat (705-035-147) were from Jackson Immuno-Research Laboratories (Cambridge, UK). Actinomycin D was from Santa Cruz Biotechnology (Dallas, TX, USA).

### 2.2. Cell Lines

Mouse fibroblast cell line NIH 3T3 (CRL-1658), rat pheochromocytoma cell line PC12 (CRL-1721), human osteosarcoma cell line U2OS (HTB-96), and human embryonic kidney-derived cell line HEK-293 were from ATCC (Manassas, VA, USA). U2OS cell lines stably transfected with wild type FGFR1 (U2OSR1) and K514R variant (U2OSR1_K514R) were kindly provided by Dr. Ellen M. Haugsten from the Department of Molecular Cell Biology, Institute for Cancer Research, Oslo University Hospital. NIH 3T3 cells were grown in Dulbecco’s modified Eagle’s medium (DMEM; high glucose, w/o sodium pyruvate; Gibco, Thermo Fisher Scientific, Waltham, MA, USA) with 10% bovine serum (Gibco, Thermo Fisher Scientific, Waltham, MA, USA). PC12 cells were cultured in RPMI 1640 medium (BioWest, Nuaillé France) supplemented with 10% horse serum (Gibco, Thermo Fisher Scientific, Waltham, MA, USA) and 5% fetal bovine serum (Gibco, Thermo Fisher Scientific, Waltham, MA, USA). U2OS and HEK-293 cells were cultured in DMEM (high glucose, with sodium pyruvate; Nuaillé BioWest, France) with 10% fetal bovine serum (Gibco, Thermo Fisher Scientific, Waltham, MA, USA). Stably transfected U2OS cell lines were grown in DMEM with 10% fetal bovine serum supplemented with 0.5 mg/mL geneticin (BioShop, Burlington, Canada). All culture media were supplemented with antibiotics (60 μg/mL penicillin and 100 μg/mL streptomycin) from BioWest (Nuaillé, France). All cell lines were cultured in a 5% CO_2_ atmosphere at 37 °C.

### 2.3. Expression and Purification of Recombinant Proteins

*Escherichia coli* strain Bl21(DE3)pLysS from Merck (Darmstadt, Germany) was used for FGF1 proteins (wild type and mutational variants) production. A pET-3c vector (Novagen, Madison, WI, USA) containing a DNA fragment encoding Met-Ala-FGF1_22–155 was used to express wild-type FGF1. FGF1 constructs with mutated NLS sequences (ΔNLS, NLS1, NLS2A, NLS1,2A [7,13] in the pET-3d vectors were obtained from GeneUniversal (Newark, DE, USA) as a custom gene synthesis. Stabilizing mutations (Q40P, S47I, H93G) [34] were introduced by QuickChange site-directed mutagenesis using mutagenesis primers (Genomed, Warszawa, Poland).Expression and purification of FGF1 variants were performed as previously described [35]. Briefly, *E. coli* Bl21(DE3)pLysS cells transformed with plasmids containing FGF1 variant sequences were cultured at 37 °C to OD_600_ 0.8 in LB medium from BioShop (Burlington, Canada) supplemented with 100 µg/mL ampicillin and 30 µg/mL chloramphenicol. Protein expression was induced by adding IPTG to a final concentration of 1 mM. After 16 h of overnight culture at 25 °C, the bacteria were harvested. The bacterial pellet was resuspended in buffer (20 mM Tris/HCl, 0.1 mM PMSF, 1 mM EDTA, 1 mM DTT, 0.5 M NaCl; pH 7.4), sonicated and centrifuged. FGF1 proteins were purified from the soluble fraction using a Heparin-Sepharose column with a linear NaCl gradient. Recombinant proteins were analyzed by SDS-PAGE and mass spectrometry. The secondary and tertiary structures of FGF1 variants were verified by circular dichroism (J-715 or J-815 spectropolarimeter; Jasco, Easton, MD, USA) and fluorescence (FP-750 or FP-8500 spectrofluorimeter; Jasco, Easton, MD, USA) measurements as previously described [34,35].

### 2.4. Transfections and Silencing

The coding sequences of FGF1 variants were also cloned into transfection vector containing the myc tag, pcDNA3.1 (Invitrogen, Thermo Fisher Scientific, Waltham, MA, USA) and later used for transient and stable transfections of cells. PC12 cells were transiently transfected with these plasmids using Lipofectamine LTX with Plus Reagent (Thermo Fisher Scientific, Waltham, MA, USA). U2OS and HEK-293 cells were transfected using FuGene HD transfection reagent (Promega, Madison, WI, USA) according to the manufacturer’s protocol. For transient transfections, cells were seeded onto plates 24 h before transfection and experiments were performed 24–72 h after transfection. Stably transfected U2OS cells were re-plated 24 h after transfection and cultured in selection medium (with additional 0.5 mg/mL geneticin) until colony formation was observed. Cloning discs were used to transfer colonies to 6-well plates, and then cells were transferred to T-75 cm^2^ flasks or experimental plates. In all cases, expression of FGF1 variants was verified by Western blotting with anti-FGF1 antibodies.

Silencing of LRRC59 in U2OSR1 cells was performed with siRNA against LRRC59 (Eurofins MWG Operon, Eserberg, Germany) [26] or non-targeting siRNA (Horizon, PerkinElmer, Boston, MA, USA) using DharmaFECT transfection reagent (Horizon, PerkinElmer, Boston, MA, USA) according to the company protocol. Experiments were performed 72 h after silencing. The level of knockdown was confirmed by Western blotting using anti-LRRC59 antibodies.

### 2.5. Subcellular Fractionation

U2OSR1 were starved for 24 h and stimulated with mutated variants and wild type FGF1 (100 ng/mL) in the presence of heparin (10 U/mL). Bafilomycin A1 (10 nM) was used as a control for inhibition of the translocation process. For fractionation into membrane, cytosolic and nuclear fractions, we used a digitonin-based method, described previously [27]. Cells incubated with FGF1 for 6 h were washed with HSLP-buffer (high-salt/low-pH buffer; 2 M NaCl, 20 mM sodium acetate; pH 4.0) to remove surface bound FGF1. Cells were permeabilized with 20 ug/mL digitonin in PBS and incubated first at 25 °C for 5 min and then on ice for a further 30 min, allowing the cytosol to be released. The solution was collected and represented the cytosolic fraction. Cell debris was lysed in lysis buffer (0.15 M KCl, 40 mM Tris, 1% Triton X-100 and 2 mM EDTA; pH 7.2), centrifuged and supernatant was determined as the membrane fraction. Insoluble material was sonicated and designated the nuclear fraction. To fractionate cells into two fractions (cytoplasmatic and nuclear), cells were washed with PBS and lysed in a lysis buffer (20 mM Tris, 150 mM NaCl, 1mM EDTA, 1% Triton X-100; pH 7.5) containing protease inhibitor cocktail for 20 min on ice. The lysates were then centrifuged for 5 min at 17,000× *g* at 4 °C.The Triton X-100 soluble fraction was collected as the cytoplasmatic fraction. The pellet (insoluble fraction) was washed, sonicated, and was used as the nuclear fraction. FGF1 proteins present in each fraction were extracted using Heparin-Sepharose resin and analyzed by SDS-PAGE. The presence of FGF1 was verified by Western blotting. Marker proteins were detected to confirm the purity of the fractions.

### 2.6. Viability and Apoptosis Measurments

Selected cells, as indicated, were subjected to various stimuli: serum deprivation, etoposide (50 µg/mL), staurosporine (1 µM), acinomycin D (5 µM), anisomycin (10 µM) in the presence or absence of specific FGFR inhibitor PD173074 (100 nM) for 24–48 h to induce apoptosis. For testing the exogenous effect, the cells were treated with FGF1 variants (100 ng/mL) and heparin (10 U/mL). PrestoBlue or AlamarBlue Cell Viability Reagent (Thermo Fisher Scientific, Waltham, MA, USA) were used to measure cell viability according to the manufacturer’s protocol. Viability was normalized and expressed as a percentage of the maximum response observed for cells treated with wild type FGF1.

To evaluate caspase3/7 activity, ApoLive-Glo Multiplex Assay (Promega, Madison, WI, USA) was used as previously described [28]. The ratio of caspase3/7 activity to cell viability was normalized toward control cells untreated or untransfected with FGFs referred to as relative caspase3/7 activity. To evaluate the mitochondrial potential of cells, the Mitopotential Assay Kit (Millipore, Merck, Darmstadt, Germany) was used according to the manufacturer’s protocol. The percentage of live, depolarized/live, depolarized/dead, and dead cells in the whole population was measured using the Muse Cell Analyzer (Millipore, Merck, Darmstadt, Germany). In addition, cells were lysed, separated by SDS-PAGE, and analyzed by Western blotting to assess the level of cleaved PARP. All experiments were performed at least three times with at least three replicates in each experiment.

### 2.7. Thermal Stability Measurments

The stability of FGF1 variants was determined from thermal denaturation curves obtained by monitoring the change in ellipticity signal at 227 nm, as described previously [35,36]. Data were analyzed using PeakFit software (Jandel Scientific Software, San Rafael, CA, USA), assuming a two-state reversible equilibrium transition.

### 2.8. FGF1-Induced Signaling and FGF1 Degradation in Cell-Conditioned Medium

FGF1-induced signaling was analyzed in serum-starved NIH 3T3 cells, which were stimulated with FGF1 variants at 10 ng/mL and 100 ng/mL, in the presence of 10 U/mL heparin for 15 min. In the next step, to assess the stability of FGF1 proteins in the cell medium, FGF1 variants were added to serum-starved NIH3T3 cells at a concentration of 100 ng/mL in the presence of 10 U/mL heparin and incubated for 48 h at 37 °C. The conditioned medium was then transferred to a new set of starved NIH 3T3 cells for 15 min, lysed, separated by SDS-PAGE, and analyzed by Western blotting. Freshly prepared FGF1 solutions at 100 ng/mL served as positive controls. In parallel, we analyzed the degradation of FGF1 variants (1 µg/mL) after 48 h in the conditioned medium in the presence of heparin (10 U/mL) by Western blotting using an anti-FGF1 antibody.

### 2.9. Statistical Analyses

The results are expressed as means ± SD. For statistical analyses, one-way analysis of variance (ANOVA) with Tukey’s posttest was applied using GraphPad Prism 5 (GraphPad Software, San Diego, CA, USA).

## 3. Results

### 3.1. Characterisation of Recombinant NLS Variants of the FGF1 Protein

**Figure 1:**
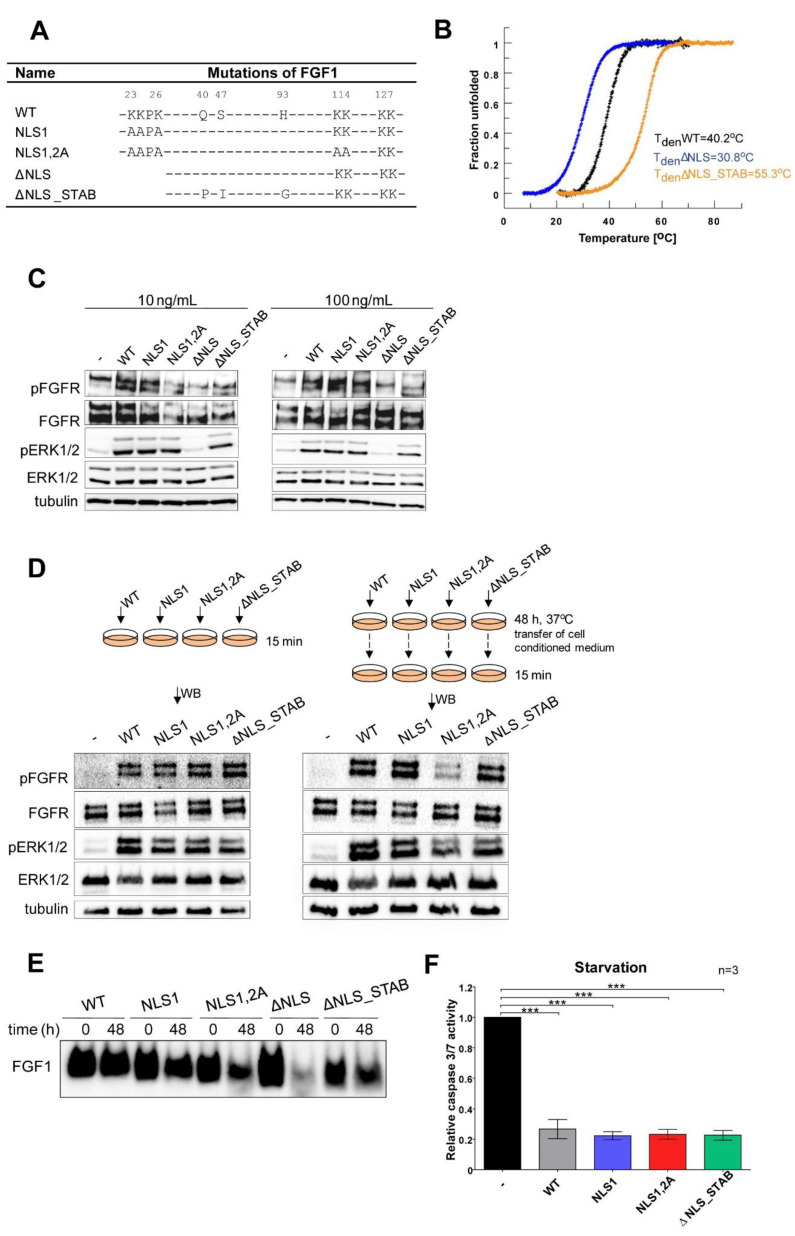
Stability and functional activity of NLS variants of FGF1. (**A**) Wild type (WT) and mutational variants of FGF1 used in the study. (**B**) Normalized thermal denaturation curves of FGF1 variants (WT, ΔNLS and ΔNLS_STAB) of FGF1 monitored by ellipticity changes at 228 nm. (**C**) The biological activity of NLS variants was assessed by analyzing the ability to activate FGF/FGFR signaling by Western blotting. Serum-starved NIH 3T3 cells were stimulated for 15 min with 10 ng/mL or 100 ng/mL of FGF1 variants in the presence of 10 U/mL heparin. (**D**) Activity of FGF1 variants after 48 h incubation with NIH 3T3 cells. Serum-starved NIH 3T3 cells were treated for 15 min with either freshly prepared 100 ng/mL of FGF1 variants and 10 U/mL heparin or cell-conditioned media after 48 h incubation with 100 ng/mL FGF1 variants in the presence of 10 U/mL heparin. Activation of downstream FGFR signaling was analyzed by Western blotting. (**E**) Degradation of FGF1 variants in cell-conditioned medium. Freshly prepared solutions of FGF1 variants (1 µg/mL) or 48 h conditioned media from NIH 3T3 cells stimulated with 1 µg/mL of the respective proteins in the presence of 10 U/mL heparin were analyzed by Western blotting using polyclonal anti-FGF1 antibodies. (**F**) For induction of apoptosis, NIH 3T3 cells were subjected to 24 h serum starvation. Then, the cells were incubated with 100 ng/mL FGF1 wild type and its mutants. After 6 h, the relative caspase 3/7 activity was determined. Data were normalized to control value and presented as means ± SD of three independent experiments. Statistical significance: *** *p* ≤ 0.001.

We have recently reported that the translocation of exogenously administrated FGF1 protects various cell lines from apoptosis independently of FGFR activation [28]. In the present study, we aimed to identify a further mechanism behind this remarkable feature. Due to several indications regarding the biological significance of the nuclear localization of FGF1, we decided to verify its role in the anti-apoptotic response. Earlier studies revealed that NLS deletion resulted in loss of protein stability, protease resistance, and affinity for FGFR [11]. Here, we analyzed a series of NLS mutational variants of FGF1 [7,13] (Figure 1A). Having at our disposal variants of FGF1 with significantly improved stability, we generated an FGF1 mutant combining an NLS deletion and three stabilizing substitutions (Q40P, S47I, and H93G) as previously described [34] named ΔNLS_STAB. Analysis of thermal denaturation curves confirmed very low stability of ΔNLS variant (T_den_=30.8 °C) as compared with the wild type (T_den_=40.2 °C). Introduction of stabilizing mutations increased the denaturation temperature of ΔNLS to 55.3 °C (Figure 1B). In the next step, we evaluated whether the NLS variants remain functional in cell culture. Thus, NIH 3T3 cells were starved for 24 h followed by stimulation with wild type FGF1 or its variants (NLS1, NLS1,2A, ΔNLS, and ΔNLS_STAB) at the concentration of 10 ng/mL and 100 ng/mL in the presence of heparin (10 U/mL). Receptor phosphorylation and activation of the MAPK signaling cascade after 15 min incubation served as the indicators of protein activity. As expected, the unstable ΔNLS variant was unable to induce FGFR and ERKs phosphorylation in contrast with the other variants (Figure 1C). Since these results confirm the loss of stability of the ΔNLS variant of FGF1 followed by loss of activity, this recombinant protein was omitted in later experiments.

We also verified how the introduced mutations affect protein degradation in the cell culture system. Activation of the MAPK signaling cascade was used to determine whether variants remained functional after incubation in culture medium. For this purpose, starved NIH 3T3 cells were stimulated with 100 ng/mL of fresh FGF1 variants (WT, NLS1, NLS1,2A, ΔNLS_STAB) in the presence of heparin (10 U/mL) or FGF1 proteins incubated for 48 h in conditioned media (Figure 1D). Western blotting analysis showed that all variants retained their ability to bind FGFR and activate FGFR and downstream signaling, as indicated by phosphorylation of ERK1/2. However, the NLS1,2A variant exhibited slightly weaker activity (Figure 1D). Furthermore, we analyzed the degradation of FGF1 variants (1 µg/mL) in NIH 3T3 conditioned medium in the presence of heparin (10 U/mL) after 48 h (Figure 1E) by Western blotting. We observed that indeed the ΔNLS variant was labile in medium and rapidly degraded within 48 h, whereas its stable version (ΔNLS_STAB) remained almost intact. As expected, the NLS1.2A variant showed increased susceptibility to degradation, which is consistent with its reduced ability to activate downstream signaling.

In the next step, we evaluated the anti-apoptotic potential of exogenously administrated FGF1 variants in NIH3T3 cells. In this experiment, apoptosis was induced by 24 h serum starvation and then 100 ng/mL of each variant was added to the cells for 6 h. After that time, caspase 3/7 activity, indicating progression of apoptosis, was measured along with cell viability. The anti-apoptotic response induced through FGFR activation and downstream signaling was virtually the same for all variants tested except for the ΔNLS mutant (Figure 1F), reflecting their ability to bind and activate the receptor. Overall, our results indicate that all FGF1 NLS variants except ΔNLS do not degrade and remain functional in the cell culture system.

### 3.2. Exogenously Added NLS Variants of FGF1 Deficient in Nuclear Translocation Retain Intracellular Anti-Apoptotic Properties

**Figure 2:**
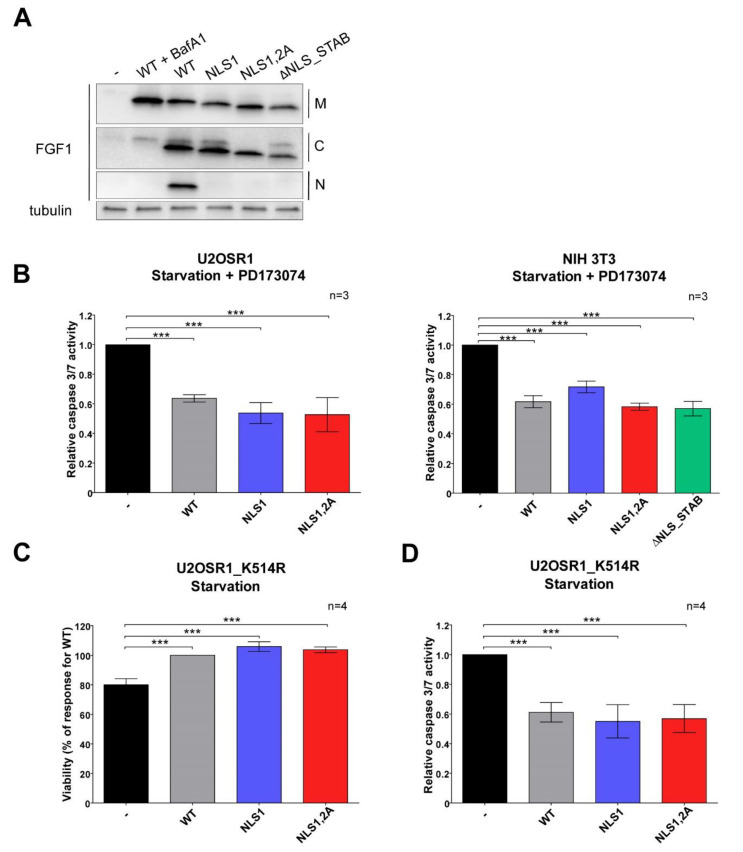
Translocated FGF1 inhibits apoptosis independently of its nuclear localization and receptor activation. (**A**) NLS variants are effectively translocated to cytosol but are defective in transport to the nucleus. The presence of FGF1 variants in the membrane (M), cytosolic (C) and nuclear (N) fractions was confirmed by subcellular fractionation after 6 h serum deprivation in U2OSR1 cells. Tubulin in the cytosolic fraction served as an equal loading control. (**B**) Anti-apoptotic activity of FGF1 variants at 100 ng/mL in serum-starved cells expressing FGFR1 (U2OSR1 and NIH 3T3) in the presence of potent chemical FGFR inhibitor (100 nM of PD17307), as determined by caspase 3/7 activity measured 16 h and 6 h after growth factors’ stimulation, respectively. (**C**,**D**) Anti-apoptotic activity of FGF1 variants at 100 ng/mL in serum-starved U2OSR1_K514R cells (U2OS cells stably expressing FGFR1 kinase-dead mutant), as determined by cell viability with Presto Blue reagent (**C**) and relative caspase 3/7 activity using ApoLive-Glo Multiplex Assay (**D**) evaluated 48 h and 16 h after growth factors’ stimulation, respectively. Data were normalized to control values and presented as means ± SD of three or four independent experiments. Statistical significance: *** *p* ≤ 0.001.

Since we had previously demonstrated that translocated FGF1 promote cell survival independently of receptor activation, we tested the competence of NLS variants in inducing an intracellular anti-apoptotic response. First, using U2OSR1 cells, we confirmed again that NLS variants of FGF1 (NLS1, NLS1,2A, and ΔNLS_STAB) are indeed not translocated to the nucleus but are present in the cytosol upon 6 h serum starvation. The endosomal proton pomp inhibitor, bafilomycin A1 (BafA1, 10 nM), which blocks transport from endosomes, was used together with wild-type FGF1 in the negative control. Using subcellular fractionation, wild-type and NLS variants of FGF1 (NLS1, NLS,1,2A and ΔNLS_STAB) were shown to be present in the membrane and cytosolic fractions, whereas only wild-type FGF1 was found in the nuclear fraction. Detection of γ-tubulin in the cytosolic fraction served as a control of equal loading (Figure 2A).

We then analyzed the anti-apoptotic activities of the translocated NLS variants. To be able to separate the intracellular response from the effect of FGFR and downstream signaling pathways activation, we measured caspase 3/7 activity in two different FGFR1-expressing cell lines, mouse NIH 3T3 fibroblasts and human U2OSR1 osteosarcoma cells in the presence of a potent chemical FGFR inhibitor [28]. Apoptosis was induced by 24 h of starvation, and then 100 ng/mL of each FGF1 variant (WT, NLS1, NLS1,2A) was added to the cells for 16 h or 6 h in the presence of PD173074 in U2OSR1 or NIH3T3 cells, respectively. In this experiment, the caspase 3/7 activity in starved cell was reduced by 40% for wild-type FGF1 and by approximately 50% for NLS variants (Figure 2B). The data obtained clearly indicate that anti-apoptotic properties of FGF1 are not related to its nuclear localization.

To ensure that the observed effect is completely independent of FGFR activation, we also used U2OS cells stably transfected with the FGFR1 kinase dead mutant K514R (U2OSR1_K514R), causing complete inactivation of FGFR signaling [28]. Apoptosis was induced in a similar manner as in FGFR-inhibited U2OSR1 cells. Again, all exogenously added FGF1 variants (WT, NLS1, NLS1,2A) were able to halve starvation-induced caspase 3/7 activity in U2OSR1_K514R cells (Figure 2C). We also examined how the anti-apoptotic response translated into cell viability. Cells subjected to 24 h starvation were treated with 100 ng/mL of each FGF1 variant for 48 h. There was no difference between the viability of U2OSR1_K514R cells treated with wild type FGF1 or its NLS variants, whereas incubation without growth factors resulted in a decrease in cell viability below 80%. These data show that NLS mutants such as the wild type counteract the progression of apoptosis (Figure 2D). These data are in full agreement with the results obtained in the presence of FGFR inhibitor, confirming that exogenously administered FGF1, as a result of its translocation, protects differentiated cells from apoptosis regardless of receptor activation and its nuclear localization.

### 3.3. Wild-Type FGF1 Retains Its Anti-Apoptotic Potential despite Being Prevented from Nuclear Translocation

**Figure 3:**
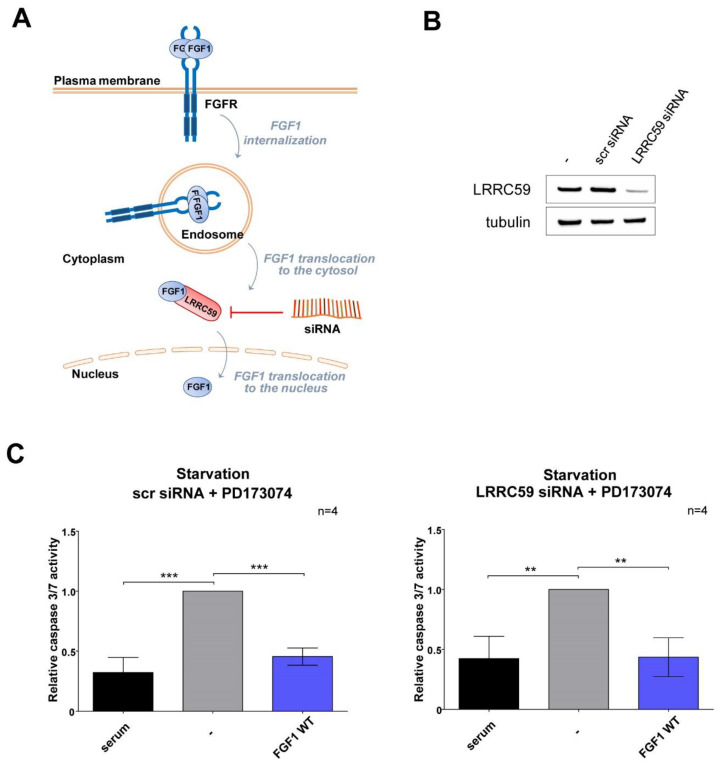
Protective effect of exogenously administrated FGF1 upon LRRC59 silencing. (**A**) Schematic of mediation of FGF1 nuclear translocation by LRRC59. (**B**) U2OSR1 cells were transfected with 100 nM of scramble siRNA or siRNA targeted LRRC59. The efficiency of LRRC59 silencing after 72 h was assessed by Western blotting. (**C**) For induction of apoptosis 72 h after silencing, cells were serum starved for 24 h, and then treated with wild-type FGF1 in the presence of FGFR inhibitor (100 nM PD173074). 16 h later, relative caspase 3/7 activity was determined. Data were normalized to control values and presented as means ± SD of four independent experiments. Statistical significance: ** *p* ≤ 0.01, *** *p* ≤ 0.001.

To verify the results obtained for NLS mutants, we also knocked-down LRRC59, a protein-mediating nuclear transport of FGF1 (Figure 3A) [22,26], in the human osteosarcoma U2OSR1 cell line constitutively expressing FGFR1. Non-targeting siRNA (scr siRNA) was used as a control. A total of 72 h after siRNA transfection, the efficiency of silencing was confirmed by Western blotting (Figure 3B). To induce apoptosis, siRNA-transfected cells were subjected to serum deprivation and treated with wild-type FGF1 (100 ng/mL) for 24 h. FGFR activation was chemically inhibited with PD173074 (100 nM) throughout the experiment allowing only the intracellular anti-apoptotic activity of translocated FGF1 to be measured. Analysis of caspase 3/7 activity analysis 16 h after FGF1 treatment showed that, independent of LRRC59 silencing, wild-type FGF1 was able to counteract apoptosis progression, reducing relative caspase 3/7 activity from 1.0 to 0.45 (Figure 3C). This finding confirms that the inability of FGF1 to undergo nuclear import does not limit its anti-apoptotic activity, at least in differentiated cells.

### 3.4. Mutations in NLS Sequence of Transiently Expressed FGF1 Do Not Affect Its Pro-Survival Response in Differentiated Cells

**Figure 4:**
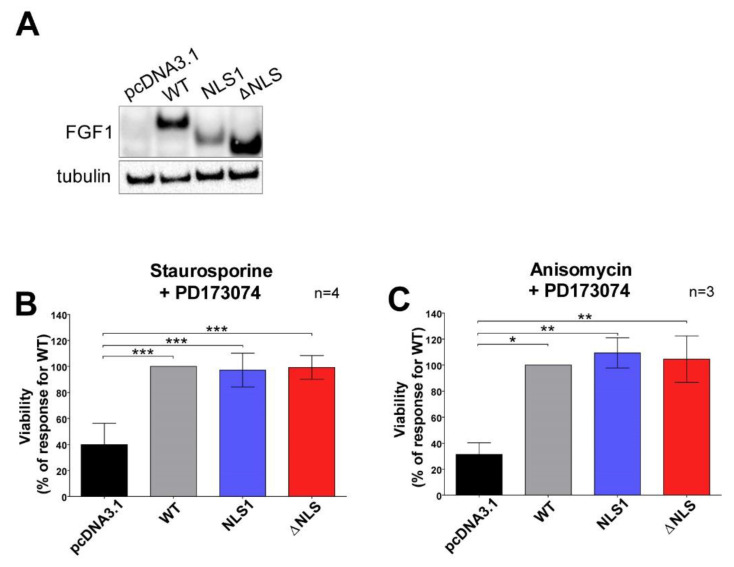
Endogenously expressed FGF1 inhibits apoptosis independently of its nuclear localization. U2OS cells were transiently transected with empty pcDNA3.1 vector or vectors encoding wild type of FGF1 (WT) and its mutated variants (NLS1 and ΔNLS). (**A**) The presence of an endogenous FGF1 pool was assessed after 48 h by Western blotting. To induce apoptosis 48 h after transfection, cells were subjected for 24 h to various apoptosis-inducing stimuli: (**B**) staurosporine (1 µM), and (**C**) anisomycin (10 µM) in the presence of the FGFR inhibitor, 100 nM PD173074. Then, the viability of cells was determined using Presto Blue reagent. Data were normalized to control values and presented as means ± SD of three or four independent experiments. Statistical significance: * *p* ≤ 0.05, ** *p* ≤ 0.01, *** *p* ≤ 0.001.

To further evaluate the impact of the NLS sequence on FGF1 anti-apoptotic activity, we transiently transfected U2OS cells with plasmids encoding wild-type FGF1 and its two mutated variants, NLS1 and ΔNLS. The empty pcDNA3.1 vector was used as a control. In this experiment, a stabilized version of the ΔNLS mutant was not used to be able to compare the results with previously published reports [33]. First, 48 h after transfection, FGF1 expression levels were analyzed by Western blotting, confirming the presence of an endogenous pool of wild-type FGF1 as well as its NLS variants (Figure 4A). Cells were then treated for 24 h with various apoptosis-inducing stimuli: staurosporine (1 µM), a potent protein kinase C inhibitor; and anisomycin (10 µM), which inhibits protein synthesis. As U2OS cells might express a very low level of FGFRs, a chemical inhibitor of FGFR (PD173074, 100 nM) was present in the culture media throughout the experiment to ensure that the observed effects originated exclusively from intracellular FGF1 proteins and not from secreted growth factors activating signaling. The anti-apoptotic properties of the FGF1 variants were assessed by measuring cell viability using Presto Blue reagent. All apoptosis inducers significantly reduced the viability of U2OS cells transfected with the empty pcDNA3.1 vector compared with FGF1-transfected cells (Figure 4B,C). Both NLS variants behaved virtually the same as wild-type FGF1, efficiently protecting cells from apoptosis. The data obtained clearly indicated that the nuclear localization of FGF1 is not crucial for its pro-survival response.

### 3.5. Stably Expressed NLS Mutants Exhibit Anti-Apoptotic Activity in U2OS Cells

**Figure 5:**
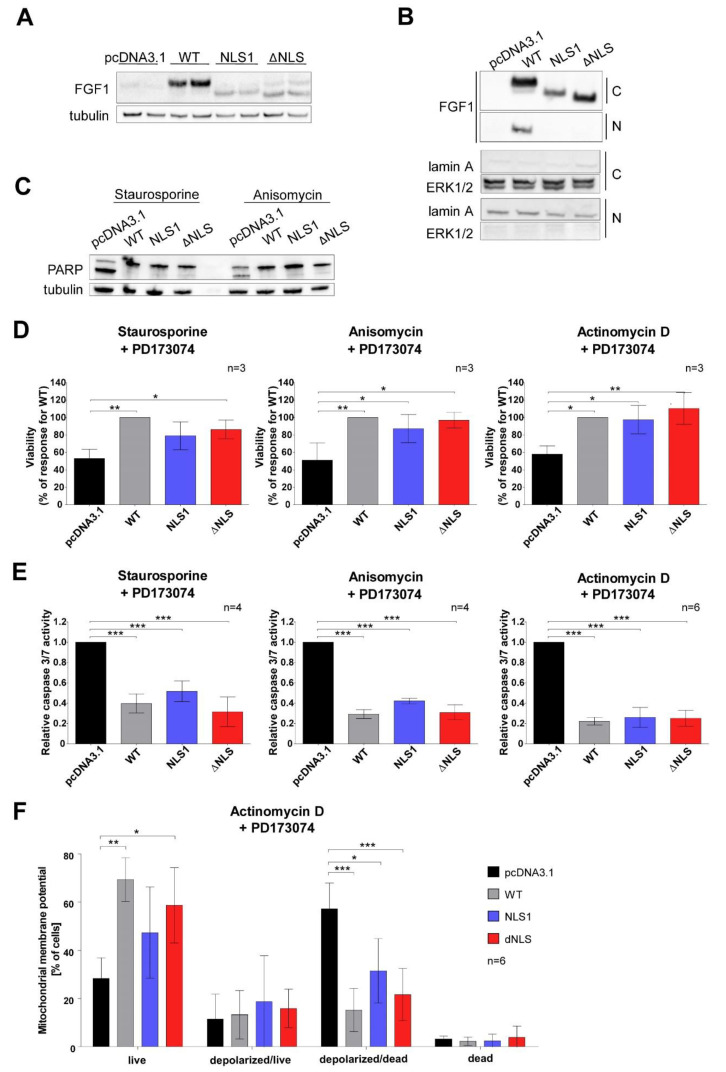
Anti-apoptotic activity of stably expressed FGF1. U2OS cells were stably transfected with vectors encoding wild type FGF1 (WT) and its mutated variants (NLS1 and ΔNLS). (**A**) The presence of pools of individual endogenous FGF1 variants in the lines was verified by Western blotting. (**B**) The presence of endogenously expressed FGF1 variants in the cytoplasmic (C) and nuclear (N) fractions was confirmed by subcellular fractionation and fraction purity was assessed by detection of the marker proteins, lamin A and ERK1/2 after 6 h serum starvation. (**C**) Cell lines were also treated for 6 h with staurosporine and anisomycin, and PARP cleavage was evaluated by Western blotting. (**D**,**E**) To induce apoptosis, cells were treated with different stimuli: staurosporine (1 µM), anisomycin (10 µM), and actinomycin D (5 µM) in the presence of FGFR inhibitor (PD173074, 100 nM). (**D**) After 24 h the viability and (**E**) caspase 3/7 activity of cells were determined. (**F**) Mitochondrial membrane potential (ΔΨm) was also measured after 24 h of actinomycin D treatment. Data were normalized to control values and presented as means ± SD from three to six independent experiments. Statistical significance: * *p* ≤ 0.05, ** *p* ≤ 0.01, *** *p* ≤ 0.001.

To confirm the above results, U2OS cell lines stably expressing FGF1 variants (WT, NLS1, ΔNLS) were also established. A cell line stably transfected with the empty plasmid pcDNA3.1 served as a control. Expression of the endogenous pool of FGF1 was routinely tested in different cell passages (Figure 5A). To verify again the lack of ability of NLS variants to translocate to the nucleus, we performed subcellular fractionation after 6 h serum deprivation (Figure 5B). We confirmed the presence of all FGF1 variants in the cytoplasmic fraction, but only the wild-type was present in the nuclear fraction, indicating that NLS1 and ΔNLS are defective in nuclear localization (Figure 5B). There was no cross contamination between subcellular fractions as determined by Western blotting of the marker proteins—lamin A for the nuclear fraction and ERK1/2 for the cytoplasmic fraction (Figure 5B).

In the next step, we evaluated the progression of apoptosis by verifying Poly (ADP-ribose) polymerase (PARP) cleavage after 6 h treatment with staurosporine or anisomycin in stably transfected U2OS cell lines. Western blotting analysis revealed that all cell lines expressing FGF1 variants were characterized by decreased PARP cleavage compared with the control U2OS cell line (Figure 5C). This further confirmed the pro-survival effects of FGF1 regardless of its nuclear localization. We also analyzed the viability of stably transfected cells subjected to various apoptosis-inducing stimuli such as: staurosporine, anisomycin, and actinomycin D, which intercalates into DNA and thus prevents the RNA polymerase progression. Cell viability was assessed after 48 h using Presto Blue reagent. In all cases, treatment with apoptosis inducers resulted in a decrease in viability of cells transfected with an empty vector (pcDNA3.1) below 60% of the viability observed for cells stably expressing wild-type FGF1 (Figure 5D). Cells expressing NLS1 or ΔNLS were characterized by high viability at similar level as wild-type transfected cells (Figure 5D). Furthermore, we determined the anti-apoptotic response in stably transfected U2OS cell lines by measuring caspase 3/7 activity 24 h after administration of various apoptosis inducers. Again, cell lines expressing all FGF1 variants (WT, NLS1, ΔNLS) exhibited significantly reduced caspase 3/7 activity (more than twofold) after treatment with staurosporine, anisomycin or actinomycin D compared with the control cell line (Figure 5E).

Since mitochondrial dysfunction (in particular, the depolarization of the ΔΨm transmembrane potential) has been shown to be involved in the induction of apoptosis, we decided to measure the mitochondrial potential of stably transfected U2OS cell lines 24 h after actinomycin D administration. It was observed that the percentage of live cells in cells expressing the WT and ΔNLS variants was significantly higher (62.0% and 56.6%, respectively) compared with control cells (23.5%), as well as the percentage of depolarized/dead cells expressing all FGF1 variants (WT—21.8%, NLS1—39.6% and ΔNLS—23.7%) was significantly lower compared with the control (61.7%) (Figure 5F). These findings indicate that the endogenous expression of all FGF1 variants reduces mitochondrial membrane permeability, preventing its depolarization and consequent release of apoptotic factors leading to cell death.

## 4. Discussion

FGF1 is a classical mitogen that leads to cell growth and proliferation through FGF receptor binding and activation. However, under stress conditions, this protein additionally has the ability to translocate into the cell interior in a receptor-mediated manner (via FGFR1 and FGFR4), but independently of FGFR activation [17,18]. For many years, the role of intracellular FGF1 was unclear. We have recently shown that translocated FGF1 is able to inhibit apoptosis and promote cell survival even when FGFR tyrosine kinase is completely inactive [28]. Despite the presence of potent FGFR inhibitors or a FGFR kinase inactivating mutation, exogenously added FGF1 is able to protect the cell from apoptosis. In accordance, the presence of translocation inhibitors (endosomal proton pumps or HSP90 inhibitors) prevents pro-survival action of FGF1 indicating that the intracellular pool of FGF1 is responsible for the anti-apoptotic effect [28]. FGF1, crossing the cell membrane, reaches the cytosol and then the cell nucleus [6]. FGF1 has been shown to contain two NLS sequences that are required for its nuclear transport [13]. In this study, we decided to verify whether the nuclear localization of FGF1 is responsible for its anti-apoptotic properties. To investigate the role of the NLS sequence in the anti-apoptotic response of FGF1, we generated a series of its mutational variants—NLS1, NLS1,2A, ΔNLS, ΔNLS_STAB.

Since the ΔNLS mutant showed lower biological activity in several previously published papers [7,8,37], we decided to check its stability and folding. The deletion of four amino acid residues from the N-terminus of FGF1 resulted in a strong reduction in its thermodynamic stability. The denaturation temperature was close to 30.8 °C, indicating that at physiological temperature the protein is almost completely unfolded. When FGF1 is not in its native conformation, the binding and translocation process may be impaired. Therefore, for the recombinant protein experiments, instead of ΔNLS mutant we decided to use its stabilized version (ΔNLS_STAB), which was generated by introduction of three stabilizing substitutions thoroughly characterized by us [34]. Increased stability of ΔNLS_STAB variant provided the proper binding and activation of the FGF receptor but did not affect its intracellular localization. In the lysine-substituted NLS mutant (NLS1), the proteins showed similar thermal stability and biological activity as the wild type [12]. The absence of the NLS sequence meant that it was not transported into the nucleus after reaching the cytosol. Using subcellular fractionation, we confirmed the results of previous work that NLS variants of FGF1 mutations (NLS1, NLS1,2A, ΔNLS_STAB) do not undergo nuclear translocation in contrast to the wild type [13].

In a subsequent step, using chemical receptor inhibition (PD17307) or a kinase dead FGFR1 mutant cell line [38,39], we found that exogenously administered NLS mutants exhibited anti-apoptotic properties in NIH 3T3 and U2OS cells expressing FGFR1 after serum deprivation, independently of receptor activation. These data strongly suggested to us that nuclear localization of FGF1 is not required for its protective effect.

We observed similar pro-survival effects in U2OSR1 cells transfected with vectors encoding NLS1 and ΔNLS and then treated with various apoptosis inducers: staurosporine, anisomycin and actinomycin D in the presence of FGFR chemical inhibitor. Using cell lines stably expressing NLS variants (NLS1 and ΔNLS) of FGF1, we confirmed their anti-apoptotic properties. NLS mutants, such as wild-type FGF1, were able to significantly improve cell viability and inhibit caspase 3/7 activity when treated with different apoptosis-inducing stimuli. Furthermore, changes in mitochondrial membrane potential were observed in cells treated with actinomycin D, reflecting the progression of apoptosis. Stable expression of NLS variants resulted in a significant reduction in the pool of depolarized/dead U2OSR1 cells. Furthermore, NLS variants prevented the proteolytic cleavage of PARP. In all these experiments, we eliminated the potential anti-apoptotic effect of FGF1 originating from receptor activation (for example due to the release of FGF1 after cell disruption) with the specific FGFR inhibitor PD17307. In conclusion, these data clearly indicate that the nuclear localization of FGF1 is not important for its pro-survival response.

The LRRC59 protein is one of the intracellular binding partners of FGF1 [22]. Furthermore, LRRC59 has been shown to be essential for nuclear import of FGF1, which is mediated by Ran GTPase and α1 and β1 karyopherins [26]. Therefore, to verify the role of nuclear localization without the use of FGF1 mutants, we decided to perform apoptosis experiments when LRRC59 was knocked-down with siRNA. After LRRC59 silencing in U2OSR1 cells, FGF1 was still able to inhibit apoptosis progression induced by starvation. This result indirectly confirms that the presence of FGF1 in the nucleus is not necessary for its protective function.

Interestingly, in the context of low thermodynamic stability, in our hands, the ΔNLS mutant retained its anti-apoptotic properties when expressed inside U2OS cells. This may be due to the fact that this variant was able to adopt the proper, native conformation, thanks to the specific chaperones and other interacting partners present in the differentiated cell.

A previous study that linked the NLS sequence to the neurotrophic activity and pro-survival response of FGF1 [33] is in contrast to our results obtained in differentiated cell models. Rodriguez-Enfedaque and co-workers showed that the transfection of undifferentiated rat pheochromocytoma PC12 cells with a vector encoding the ΔNLS variant of FGF1 resulted in the inhibition of p53-dependent protection against cell death, and neurothrophic activity compared with the wild type [33]. The fact that deletion of the NLS sequence results in a significant loss of protein stability and protein unfolding may explain this discrepancy and impaired biological activity of the ΔNLS variant in some cell types [11], especially undifferentiated cells.

As our data are in conflict with results previously obtained in PC12 cells [33], we decided to include this cell line in our study. PC12 cells were transfected with vectors encoding the NLS1 or ΔNLS variant and the progression of apoptosis induced by etoposide treatment was evaluated. We obtained the same results as in Renaud’s group—with both NLS variants, cell viability was reduced, and caspase 3/7 activity was increased compared with cells transfected with wild-type FGF1 (Supplementary Figure S1A).

We assumed that the difference between the data obtained might be due to the nature of the various cell lines at different levels of differentiation. Therefore, we repeated the experiment performed for PC12 cells using a second undifferentiated cell line, human embryonic kidney cells HEK-293 (Supplementary Figure S1B), and again, the data obtained confirmed the previous results, showing that FGF1 transport to the nucleus is essential for its anti-apoptotic activity. Since we have shown that the nuclear localization of FGF1 (whether translocated or ectopically expressed) is not responsible for its anti-apoptotic response in differentiated cells, such as human osteosarcoma (U2OS) and mouse fibroblasts (NIH 3T3), we hypothesize that the mode of action of FGF1 is more complex and may vary depending on the cell type it affects. It also does not appear to be dependent on the level of FGFR receptors, nor the pool of endogenous FGF1, which was below the detection level in all cells analyzed (Supplementary Figure S2A,B). It is also possible that the observed differences between differentiated and undifferentiated cells in their response to the labile ΔNLS variant are due to the different expression levels of the FGF1 partner proteins and thus the different stability of their complexes with the growth factor.

Taking together, our results indicate that nuclear localization of FGF1 is not critical for its intracellular anti-apoptotic activity in differentiated cells and suggest that the mechanism of stress response varies with the level of cell differentiation. The exact mechanism of the anti-apoptotic activity of FGF1 is still unknown. However, there are indications that intracellular binding partners of FGF1, such as p53, may regulate its intracellular activity [30,33]. It is highly likely that interactions with other proteins involved in apoptosis, located in the cytoplasm, are responsible for the anti-apoptotic properties of FGF1. Since targeting the FGF/FGFR axis might be promising for both regenerative medicine and cancer therapies [40], our findings highlight the complexity of the mechanism of FGF1’s protective action and the need for its further investigation.

## Supplementary Materials

The following are available online at https://www.mdpi.com/article/10.3390/cells11030522/s1, Figure S1 FGF1 anti-apoptotic activity depending on its nuclear localization in undifferentiated cells. Figure S2 Characteristics of the cell lines used in the study.

## Abbreviations

BafA1	Bafilomycin A1
FGF1	fibroblast growth factor 1
FGFR	fibroblast growth factor receptor
NLS	nuclear localization sequence
NES	nuclear export sequence
PARP	Poly (ADP-ribose) polymerase
WT	wild type
