# Supplementary material for: Nuclear Localization Sequence of FGF1 Is Not Required for Its Intracellular Anti-Apoptotic Activity in Differentiated Cells
Source: Cells. 2022 Feb 2;11(3):522. doi: 10.3390/cells11030522 (PMC8833943; doi:10.3390/cells11030522)
Supplement: Supplementary file 1 [file cells-11-00522-s001.zip › cells-1545167-supplementary.pdf]

Supplementary Materials

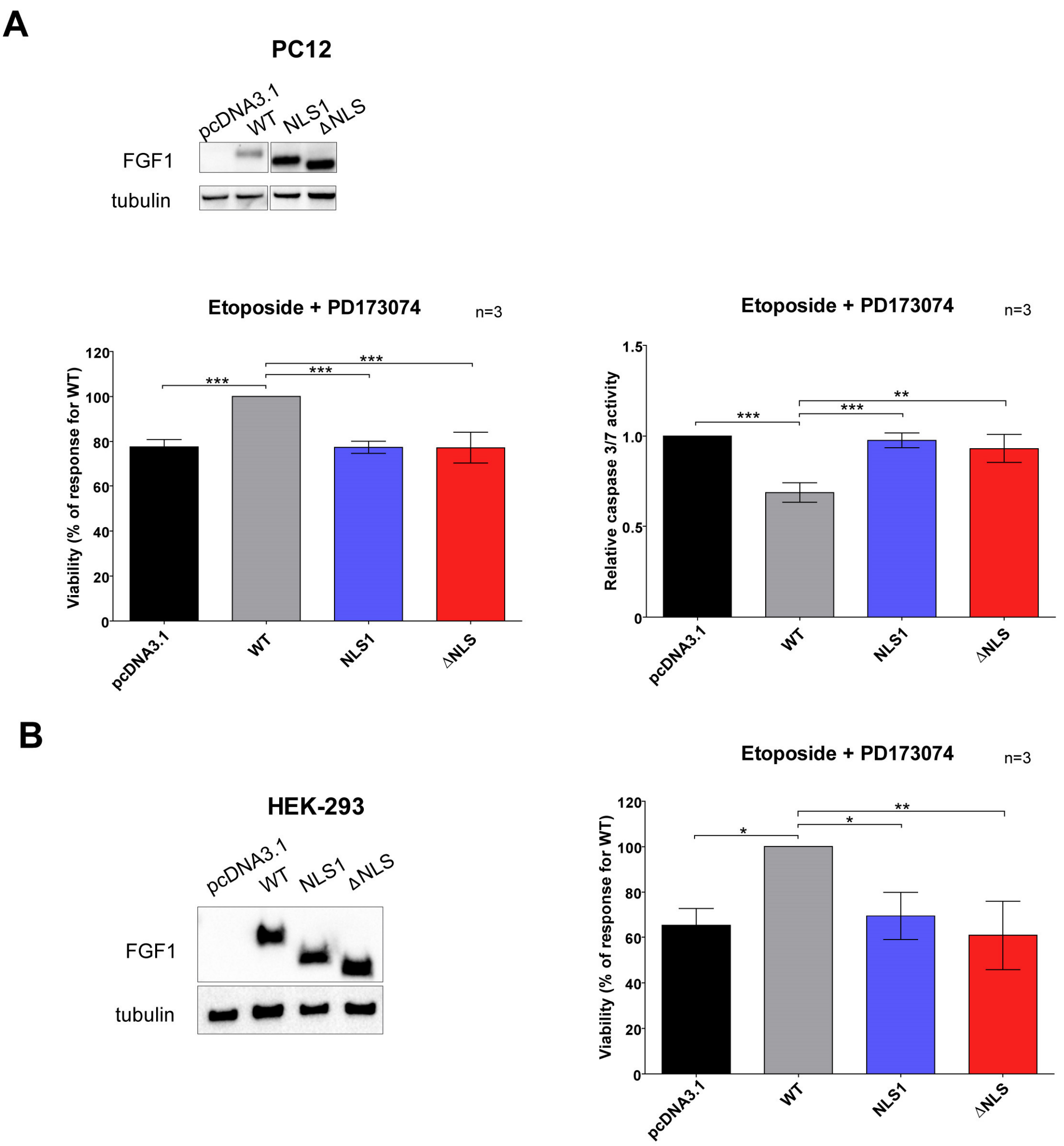

Supplementary Figure S1. FGF1 anti-apoptotic activity depending on its nuclear localization in undifferentiated cells. Confirmation of endogenous pool of FGF1 variants 48 h after transfection of (A) PC12 and (B) HEK-293 cell lines by Western blotting. 48 h post transfection cells were treated with 50 μg/mL of etoposide in the presence of FGFR inhibitor, 100 nM PD173074. The viability of cells and the caspase 3/7 activity were determined after 48 h and 24 h using Alamar Blue reagent and ApoLive-Glo Multiplex Assay, respectively. Data were normalized to control values and presented as means ± SD from three independent experiments. Statistical significance: $*p \leq 0.05$, $**p \leq 0.01$, $***\ p \leq 0.001$.

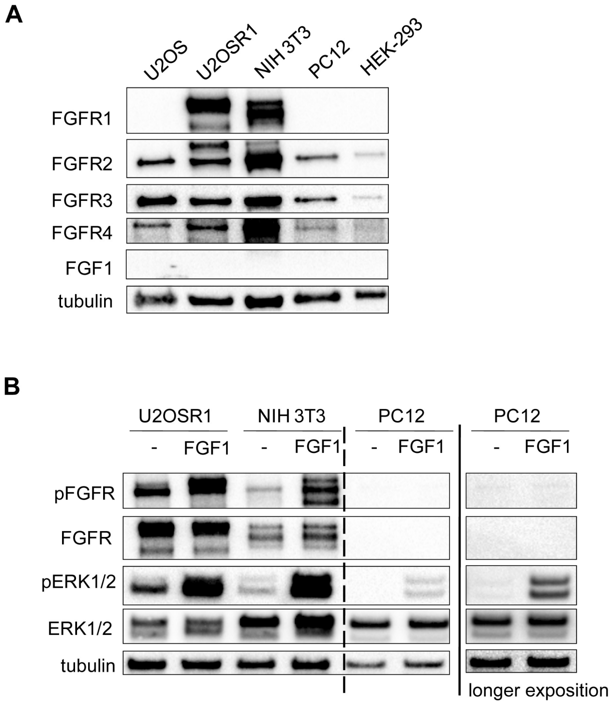

Supplementary Figure S2. Characteristics of the cell lines used in the study. (A) Analysis of FGFR1-4 expression and endogenous FGF1 pool in selected cell lines. (B) Comparison of FGFR and downstream signaling cascade ERK1/2 activation after 15-min treatment with FGF1 (100 ng/mL) in the presence of heparin (10 U/mL).
